# Supplementary material for: Observation of 1H-1H J-couplings in fast magic-angle-spinning solid-state NMR spectroscopy
Source: Nat Commun. 2024 Dec 30;15:10799. doi: 10.1038/s41467-024-55126-9 (PMC11686346; doi:10.1038/s41467-024-55126-9)
Supplement: Supplementary file 1 — Supplementary Information [file 41467_2024_55126_MOESM1_ESM.pdf]

Observation of ^{1}H-^{1}H J-couplings in fast magic-angle-spinning solid-state NMR spectroscopy

Daria Torodii,[1] Jacob B. Holmes,[1] Kristof Grohe,[2] Rodrigo de Oliveira Silva,[3] Sebastian Wegner,[2] Dimitrios Sakellariou,[3,*] Lyndon Emsley[1,*]

[1] Institut des Sciences et Ingénierie Chimiques, École Polytechnique Fédérale de Lausanne (EPFL), CH-1015 Lausanne, Switzerland

[2] Bruker BioSpin GmbH & Co KG, 76275 Ettlingen, Germany

[3] KU Leuven, M2S, cMACS, Celestijnenlaan 200F, 3001 Leuven, Belgium

Supporting Information

Raw data statement. All data and codes used are available from https://doi.org/10.5281/zenodo.14186567 under the license CC-BY-4.0 (Creative Commons Attribution-ShareAlike 4.0 International).

Table of Contents

1. **Experimental Details** 3
2. **Additional NMR data** 5
3. **Fitting procedure** 14

1. Experimental Details

All the raw data are available at: DOI: 10.5281/zenodo.14186568. The exact pulse sequences and full parameter sets used are available with the raw data.

(1S)-(−)-camphor sample was purchased from Alfa Aesar and used without further recrystallization. The powder was packed in 0.7- and 0.4-mm rotors after being crushed with a mortar and pestle.

All the 1D and 2D JRES spectra between 100 and 169.880 kHz MAS were acquired on an 18.8 T Bruker Avance Neo spectrometer corresponding to a 1H frequency of 800 MHz using a Bruker 0.4 mm HCN CP-MAS probe. The sample temperature was regulated to 298 K using VT flow. At each MAS rate, after temperature stabilization, the magic-angle was reset by maximizing signal intensity in a 1D 1H experiment using a spin-echo sequence with echo delays equal to 15 ms. The spinning was controlled by Bruker MAS III unit. The MAS instability was estimated to be roughly ± 100 Hz at all MAS rates on the 0.4 mm probe.

The refocused INADEQUATE, UC2QFCOSY and BABA-xy16 spectra of camphor at 100 kHz MAS were acquired on a 21.14 T Bruker Avance Neo spectrometer corresponding to a 1H frequency of 900 MHz using a Bruker 0.7 mm room temperature HCN CP-MAS probe. The temperature was regulated to 295 K using a VT flow at 285 K. The magic angle was optimized directly on the sample by maximizing the T2'. The spinning was controlled by Bruker MAS III unit. The MAS instability at 100 kHz MAS on the 0.7 mm probe was estimated to be roughly ± 100 Hz.

A States-TPPI acquisition scheme was used in all 2D experiments to obtain phase-sensitive two-dimensional spectra. All spectra were phase and baseline corrected. No window functions were applied prior to Fourier transformation.

Supplementary Table 1. Experimental details of the 1D ^{1}H VMAS datasets acquired for camphor with the 0.4 mm probe.

MAS rate, kHz	VT gas temperature(K)	RF amplitude (kHz)	recycle delay(s)	Acquisition time, ms	SW (kHz)	Size of real spectrum:	Number of scans
100	289.7	222.222	3	81.92	100	32768	4
108.571	288.2	222.222	3	81.92	100	32768	4
120	286.1	222.222	3	81.92	100	32768	4
140	282.1	222.222	3	81.92	100	32768	4
151.429	278.2	222.222	3	81.92	100	32768	4
160	276.1	222.222	3	81.92	100	32768	4
169.880	283.1	222.222	3	81.92	100	32768	4

Supplementary Table 2. Experimental details of the ^{1}H-^{1}H 2D JRES VMAS datasets acquired for camphor with the 0.4 mm probe.

MAS rate (kHz)	VT (K)	90° RF amplitude (kHz)	recycle delay (s)	Number of FID points: F2/F1	SW (kHz): F2/F1	Size of real spectrum: F2/F1	z-filter delay (μs)	Number of scans
100	289.7	222.222	3	32768/1024	200/1.428571	65536/2048	3	8
108.571	288.2	222.222	3	32768/1024	200/1.428571	65536/2048	3	8
120	286.1	222.222	3	32768/1024	200/1.428571	65536/2048	3	8
140	282.1	222.222	3	32768/1024	200/1.428571	65536/2048	3	8
151.429	278.2	222.222	3	32768/1024	200/1.428571	65536/2048	3	8
160	276.1	222.222	3	32768/1024	200/1.428571	65536/2048	3	8
168.571	283.1	222.222	3	32768/1024	200/1.428571	65536/2048	3	8

Supplementary Table 3. Experimental details of the ^{1}H-^{1}H 2D J- and dipolar-based spectra acquired for camphor at 100 kHz MAS with the 0.7 mm probe.

2D experiment	VT (K)	90° RF amplitude (kHz)	recycle delay (s)	Number of FID points: F2/F1	SW (kHz): F2/F1	Size of real spectrum: F2/F1	Echo delay (ms)	Number of scans
Refocused INADEQUATE	285	285.714	2	4096/ 1024	10/5	8192/2048	10	32
UC2QFCOSY	285	294.118	2	4096/ 484	9.090909/5	8192/1024	10	64
BABA-xy16	285	285.714	4.625	4096/ 512	9.090909/ 10	8192/1024	-	16

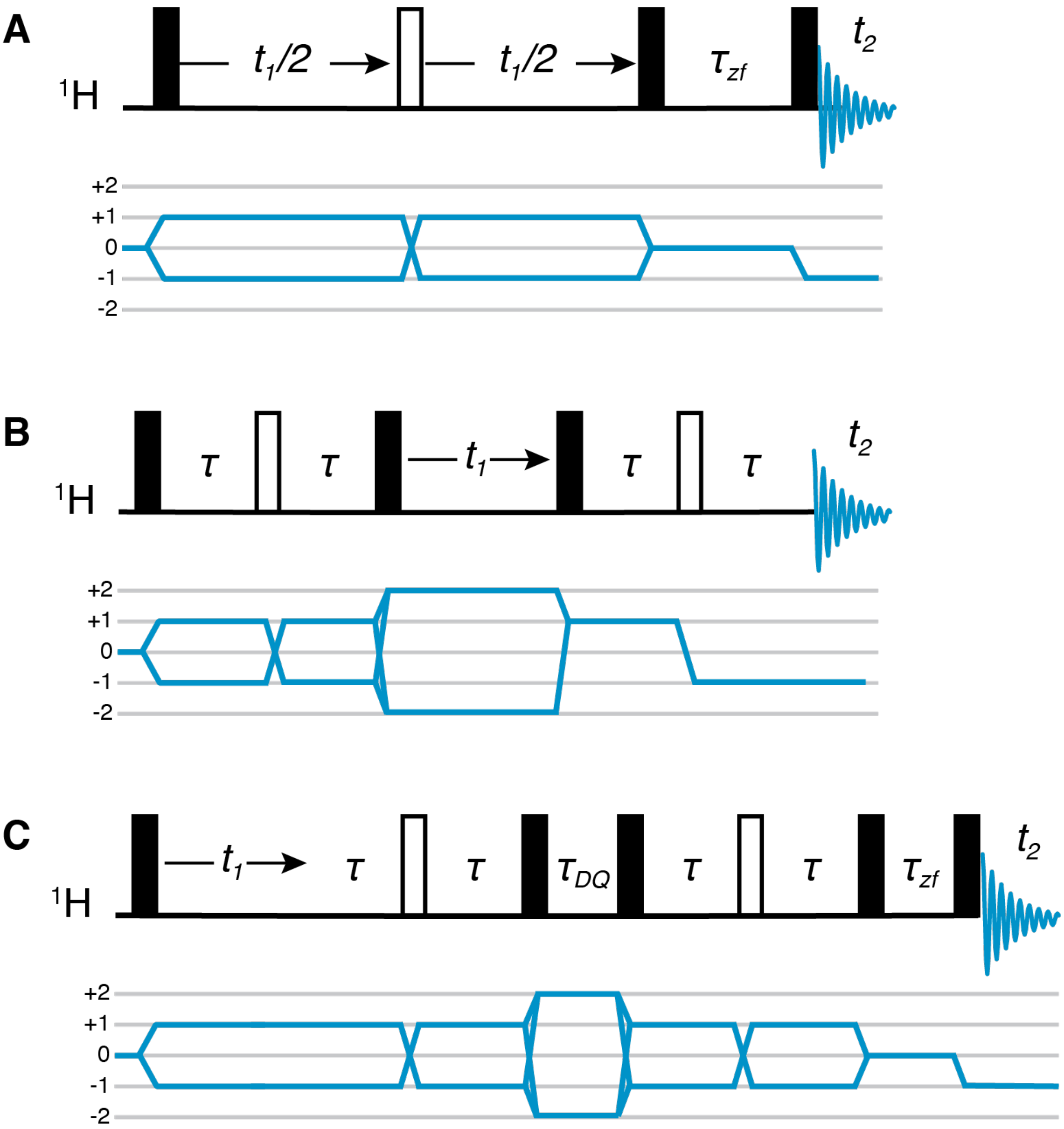

Supplementary Figure 1. A) 2D JRES, **B)** refocused INADEQUATE, **C)** UC2QFCOSY pulse sequences and coherence transfer pathways. The black filled rectangles represent 90° pulses. The unfilled rectangles represent 180° pulses.

2. Additional NMR data

Supplementary Table 4. ^{1}H-^{1}H J-coupling constants of camphor measured in DMSO-d$_6$ solution.

J, Hz	H3'	H4	H5'	H3	H6'	H6	H5
H3'		4.7	3.2	18.1			
H4	4.7		4.5				
H5'	3.2	4.5			12.0	4.0	11.3
H3	18.1						
H6'			12.0			12.3	~ 3.5
H6			4.0		12.3		~ 11.5
H5			11.3		~ 3.5	~ 11.5	

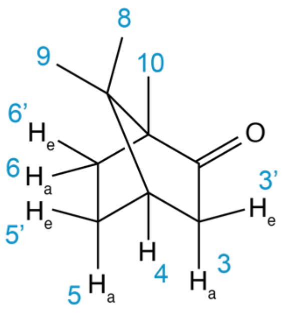

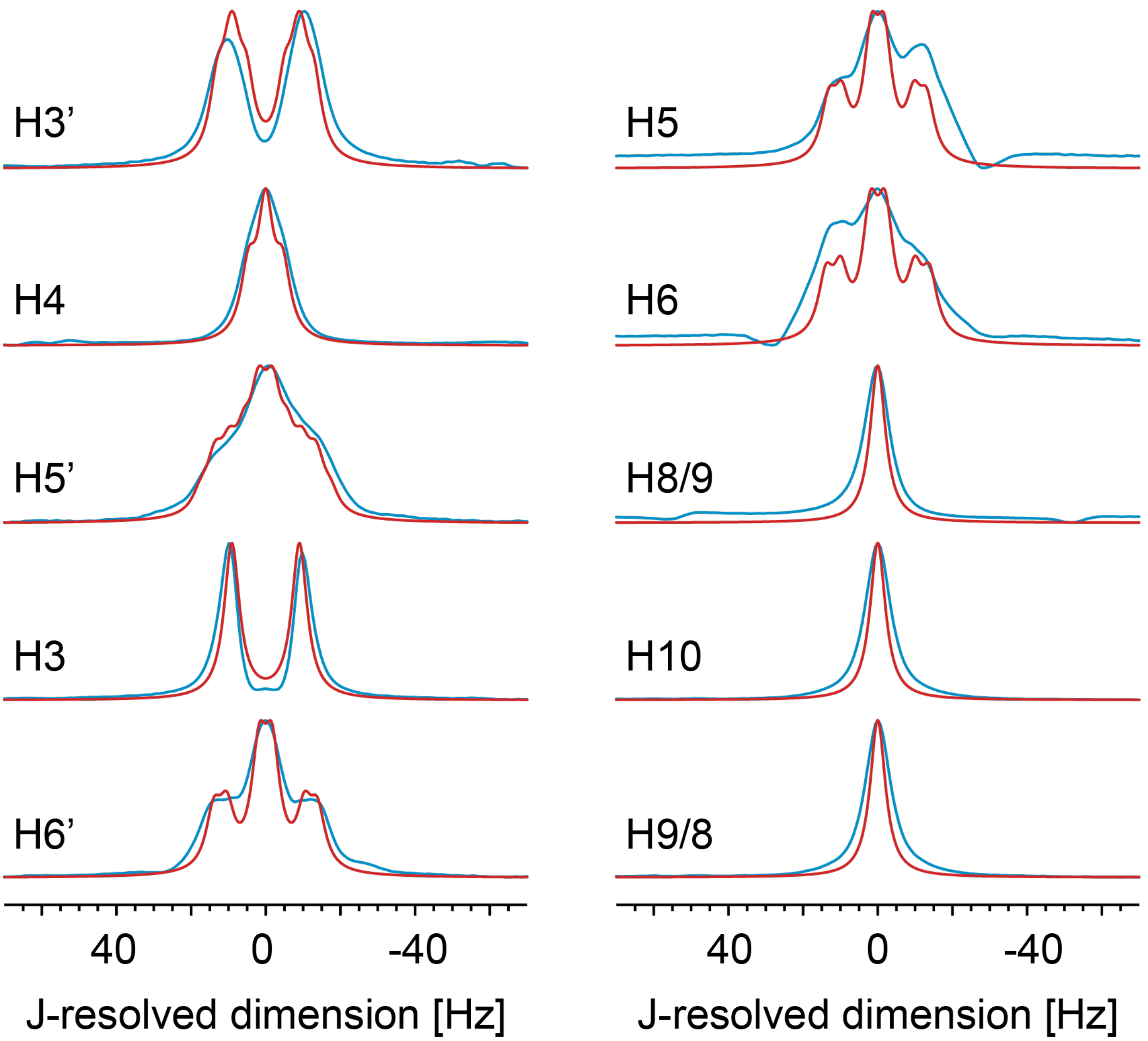

Supplementary Figure 2. Columns extracted from the 800 MHz ^{1}H-^{1}H 2D JRES spectrum of camphor acquired at 168.571 kHz MAS in blue overlaid with a simulated purely Lorentzian peaks having a linewidth of 5 Hz and subject to all J-splittings measured for camphor in solution NMR (Table S4) in red.

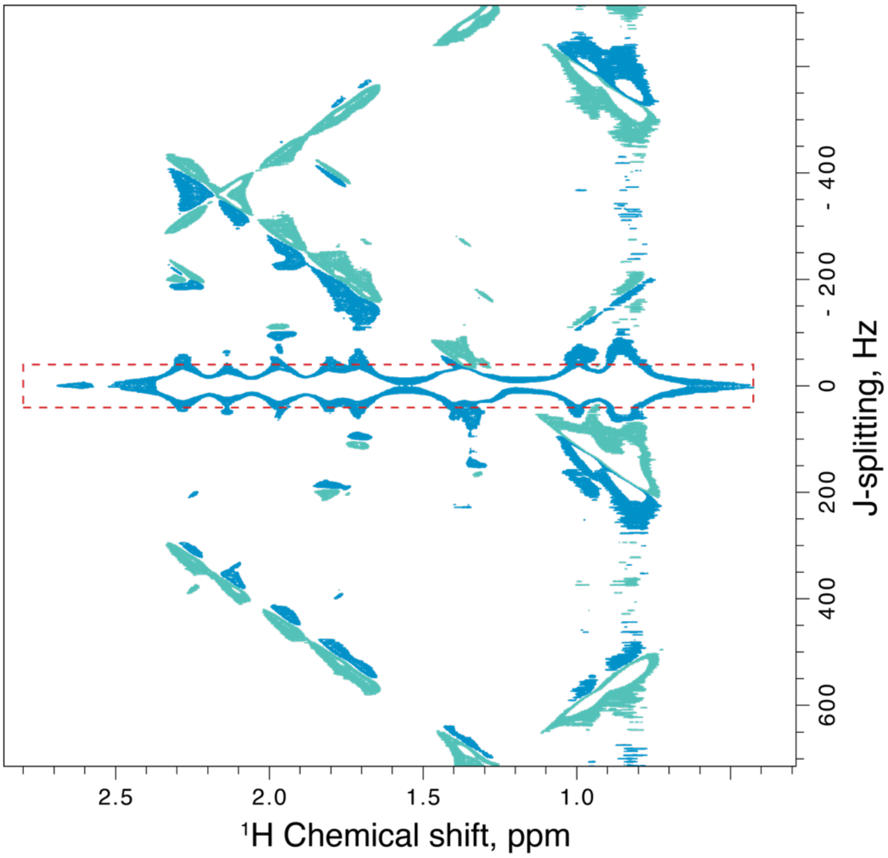

Supplementary Figure 3. 800 MHz 2D JRES spectrum at 168.571 kHz MAS showing the full ω_1 dimension. The spectrum is plotted at a contour level such that the folded artefacts mentioned in the main text are visible. The red dashed rectangle corresponds to the spectral limits of the 2D JRES spectrum shown in Figure 1B.

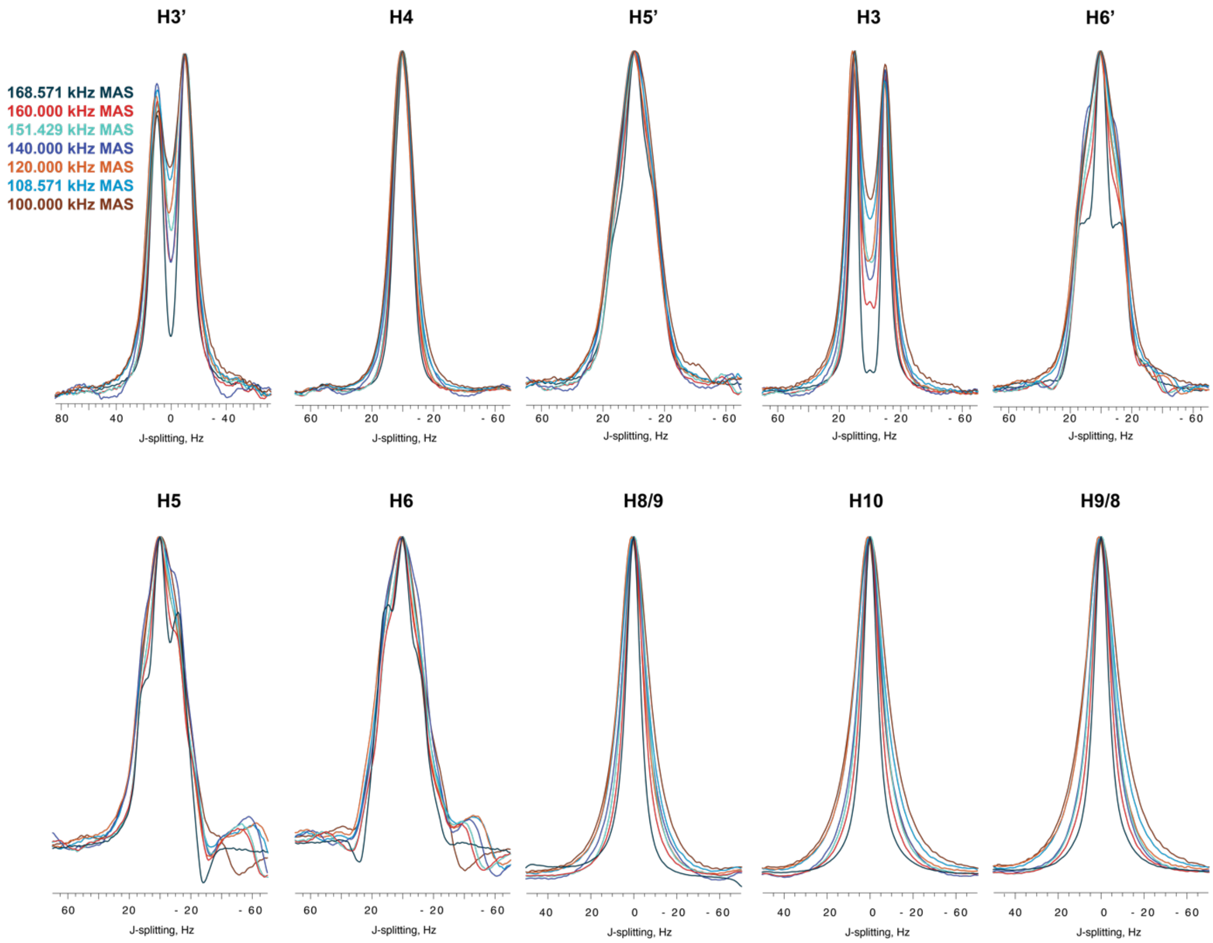

Supplementary Figure 4. Extracted columns from 800 MHz 2D JRES spectra at 100-168.157 kHz MAS.

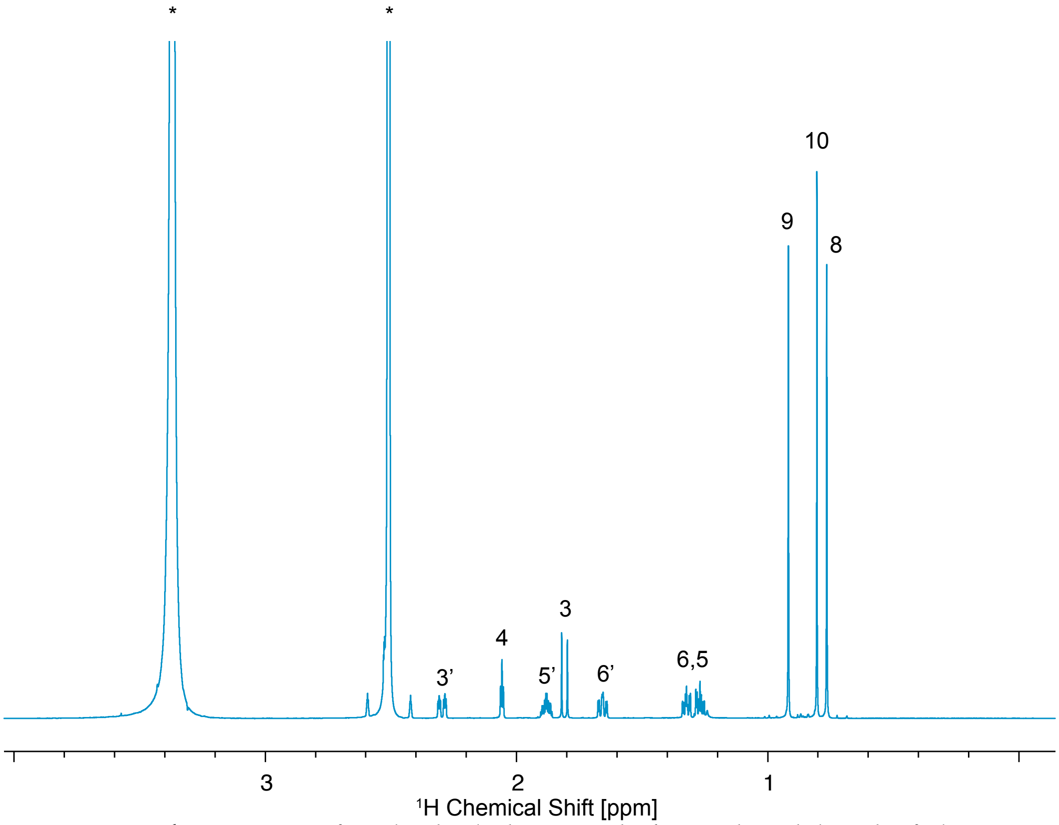

Supplementary Figure 5. ^{1}H 1D spectrum of camphor dissolved in DMSO-d_6. The asterisks mark the peaks of solvent trace impurities.

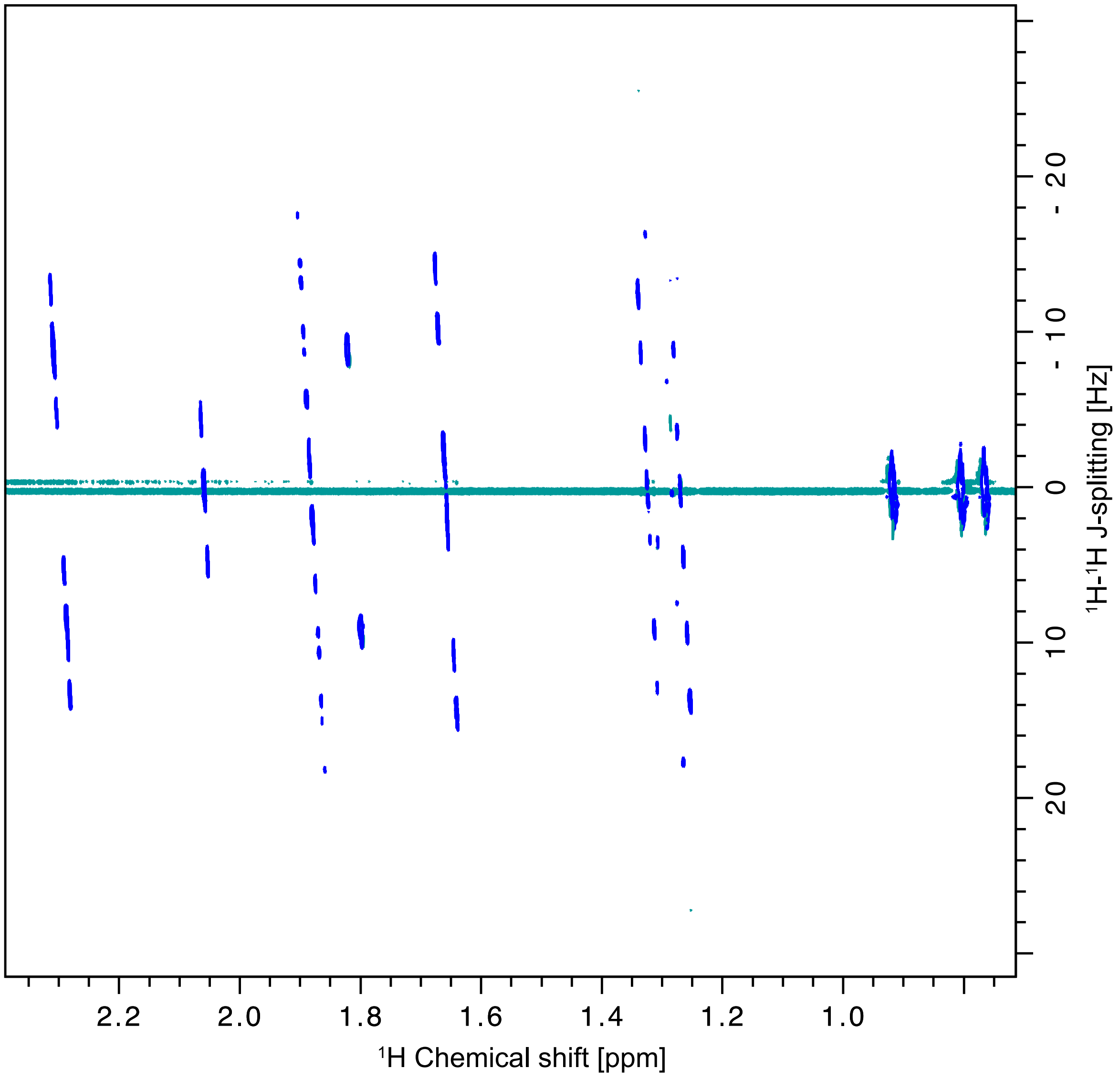

Supplementary Figure 6. ^1H-^1H phase sensitive 2D JRES spectrum of camphor dissolved in DMSO-d_6 using the Bruker pulse sequence jresgpph.

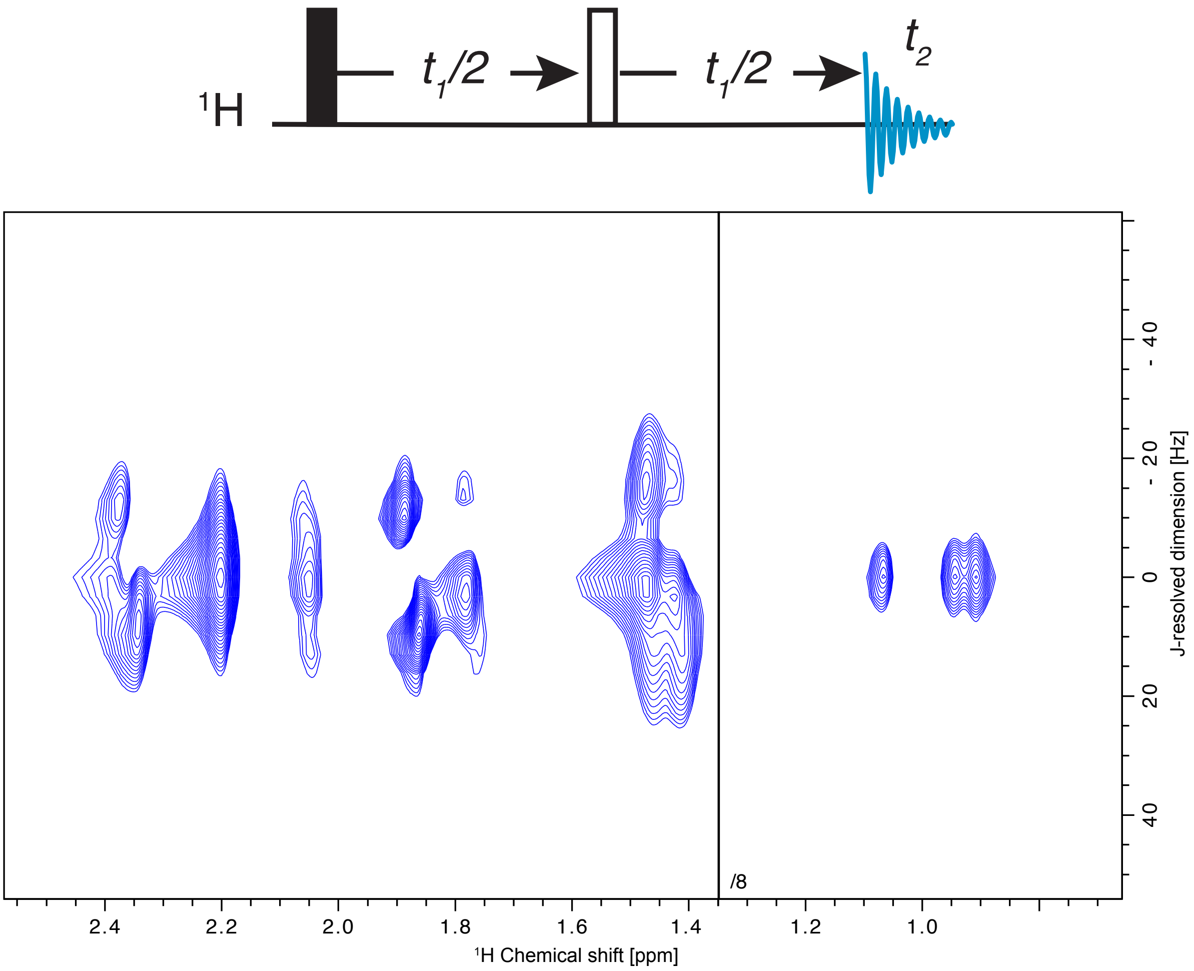

Supplementary Figure 7. Contour plot of a 900 MHz 2D JRES spectrum shown in magnitude mode of camphor at 100 kHz MAS acquired using the Bruker pulse sequence jresqf modified to make the echo delays rotor synchronized and to add a presaturation block. The phase cycling was the same as in jresqf. The pulse sequence is shown above the spectrum. The black filled rectangle represents a 90° pulse. The open rectangle represents a 180° pulse.

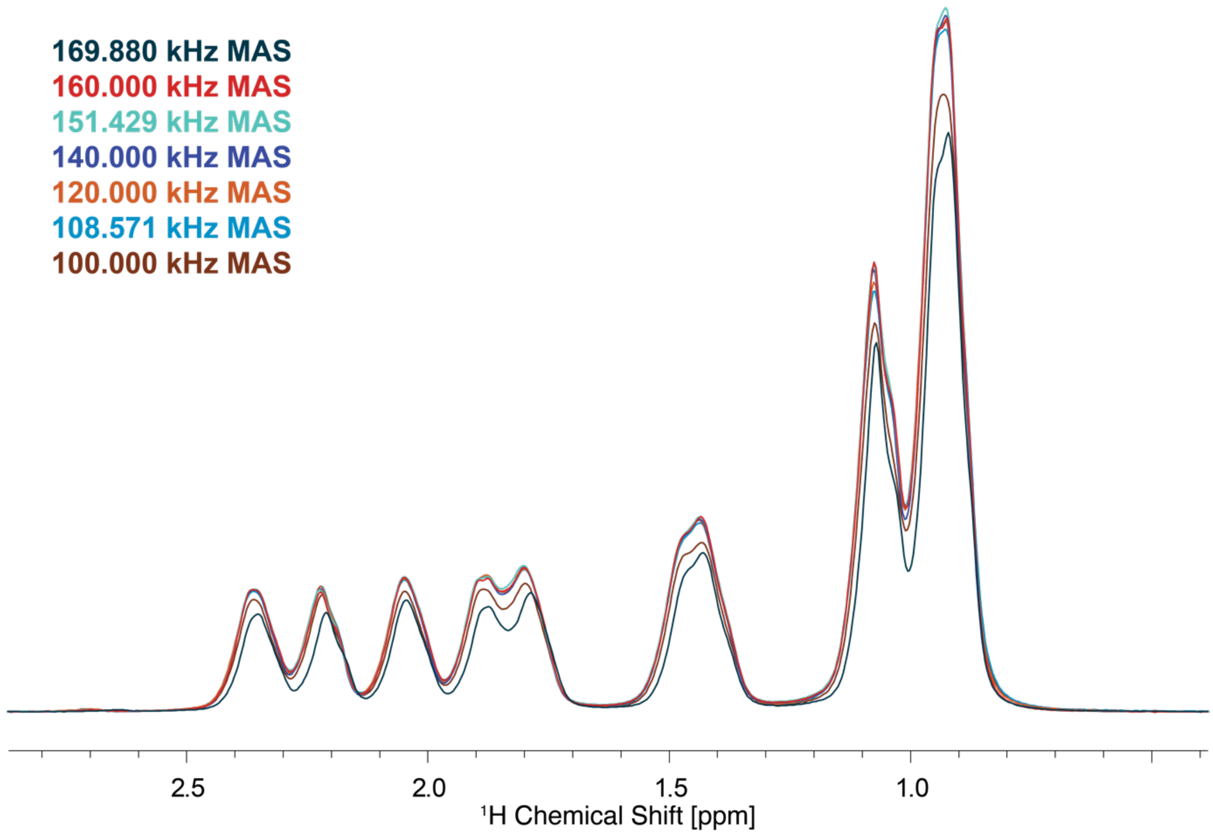

Supplementary Figure 8. 800 MHz 1D ^{1}H spectra of camphor acquired at 100-169.880 kHz MAS. In the 1D ^{1}H spectra of camphor, the linewidth is dominated by the inhomogeneous interactions, notably structural disorder and ABMS (as well as any B_0 field inhomogeneity). Altogether, these MAS independent contributions represent roughly 75% of the total ^{1}H 1D linewidth.

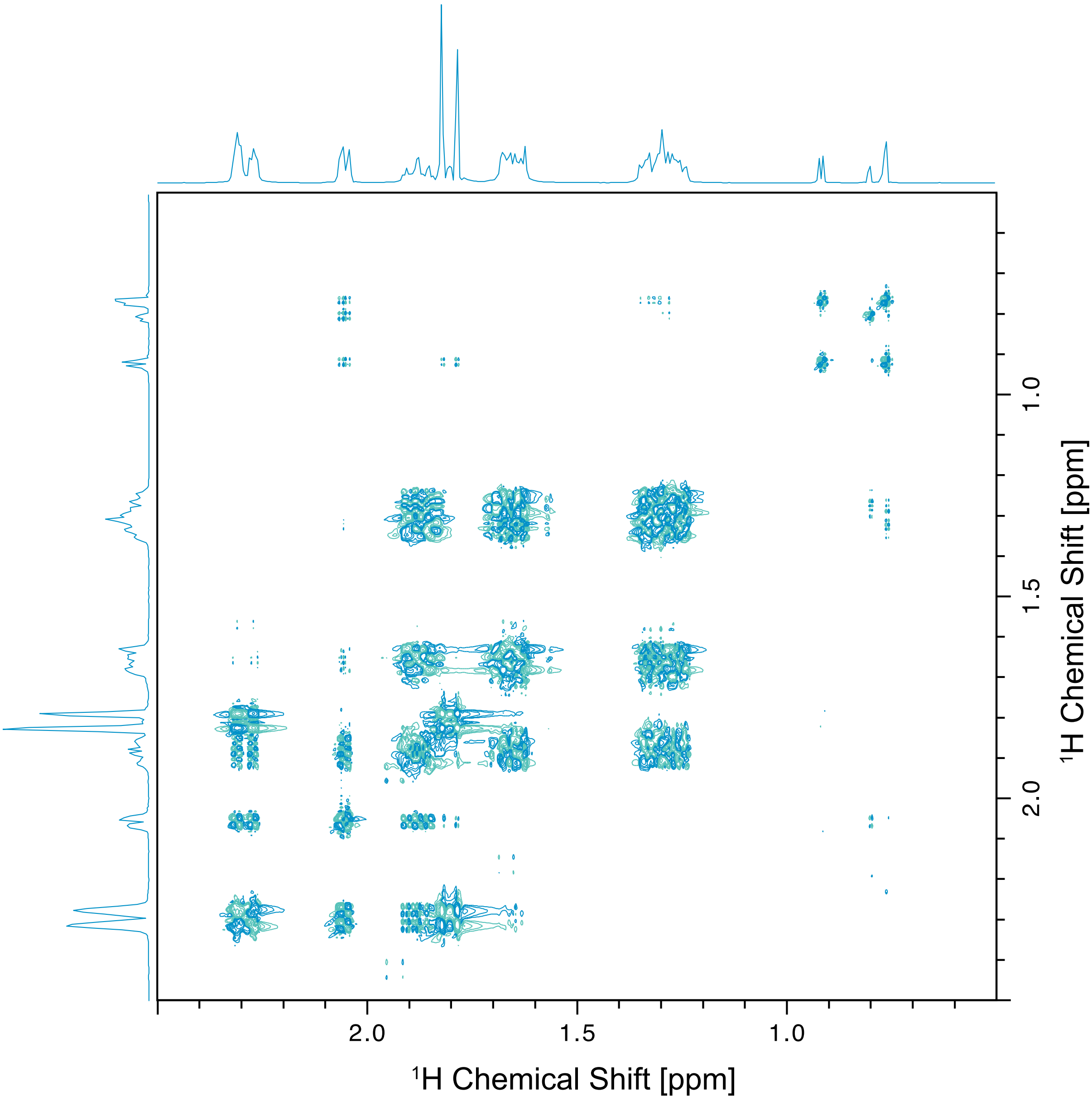

Supplementary Figure 9. 500 MHz ^{1}H-^{1}H 2D DQF-COSY spectrum of camphor dissolved in DMSO-d_6.

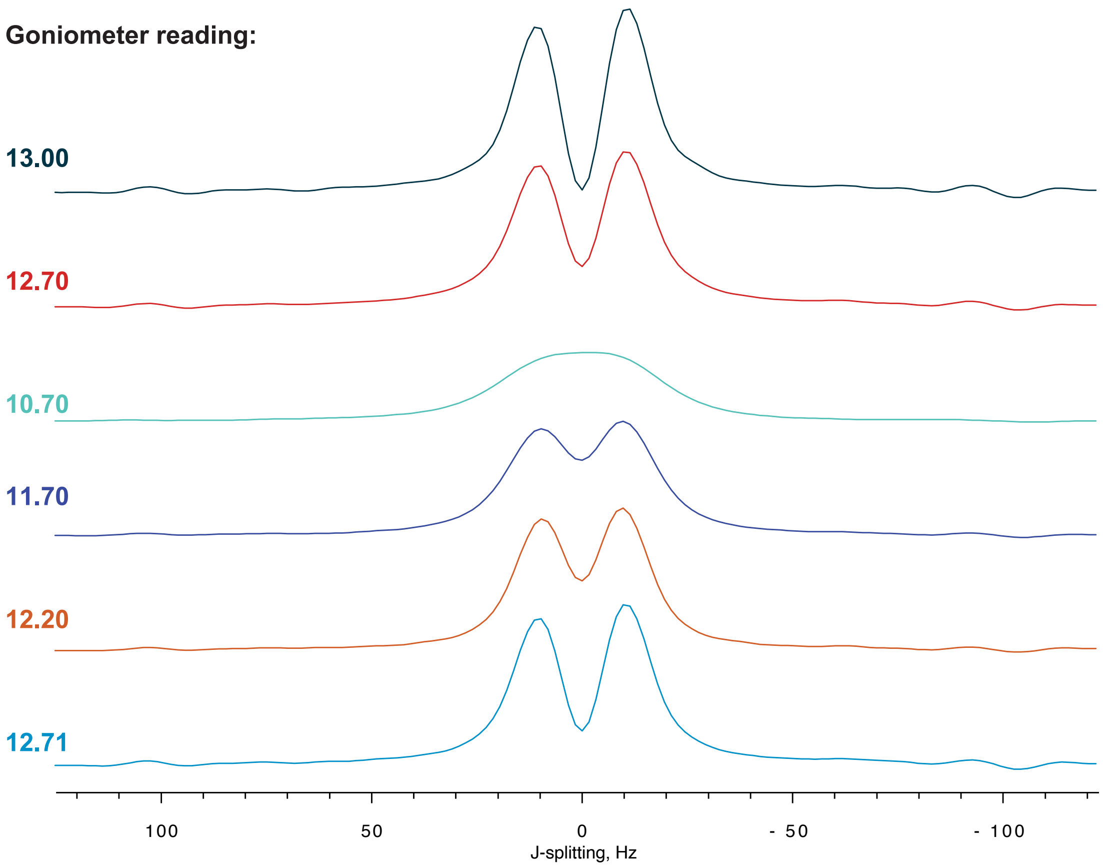

Supplementary Figure 10. Extracted H3' columns from 900 MHz 2D JRES spectra at 100 kHz MAS on the 0.7 mm HCN probe with variable setting of the magic-angle. The reading of the goniometer is given in the figure.

3. Fitting results

The complete description of the fitting protocol of the 2D JRES columns is given in the Mathematica notebook Camphor_Solids_LW_clean.nb. The results are given in Tables S4 and S5.

The distribution of the ^1H-^1H dipolar couplings was estimated in the Mathematica notebook Camphor Dipolar calculation.nb.

Supplementary Table 5. 800 MHz ^1H-^1H 2D JRES fitted linewidths by column.

MAS rate, kHz	H3' fitted linewidth, Hz	H3' linewidth fitting error, Hz	H3 fitted linewidth, Hz	H3 linewidth fitting error, Hz
168	9.4	0.5	5.2	0.4
160	11.1	0.4	7.1	0.4
151.429	12.8	0.4	9.3	0.5
140	12.0	0.6	9.1	0.4
120	14.0	0.3	10.2	0.2
108.571	14.8	0.5	11.5	0.6
100	16.2	0.5	12.8	0.5

Supplementary Table 6. 800 MHz ^1H-^1H 2D JRES columns fitted J-coupling constants.

MAS rate, kHz	J-splitting obtained by fitting H3', Hz	J fitting error, Hz	J-splitting obtained by fitting H3, Hz	J fitting error, Hz
168	21.4	0.5	20.2	0.4
160	20.6	0.4	20.0	0.4
151.429	20.6	0.4	19.9	0.5
140	21.6	0.6	20.1	0.4
120	21.1	0.3	20.8	0.2
108.571	20.1	0.5	19.6	0.6
100	20.1	0.5	20.0	0.5
